# Primary Tumor Epigenetic and Transcriptomic Alterations Associated with Nodal Burden and Metastatic Risk in ER+/HER2− Breast Cancer

**DOI:** 10.3390/ijms27177901

**Published:** 2026-09-04

**Authors:** Sandra Iñiguez-Muñoz, Andrés F. Bedoya-López, Miquel Ensenyat-Mendez, Pere Llinàs-Arias, Sookyung Ahn, Rachel E. Factor, Diego M. Marzese, Maggie L. DiNome

**Affiliations:** 1 Department of Surgery, Duke University School of Medicine, Durham, NC 27710, USA; sandra.iniguez@idisba.es (S.I.-M.); miquel.ensenatmendez@duke.edu (M.E.-M.); sooahn79@gmail.com (S.A.); 2 Cancer Epigenetics Laboratory, Health Research Institute of the Balearic Islands (IdISBa), 07120 Palma, Spain; andresfelipe.bedoya@idisba.es (A.F.B.-L.); pere.llinas@idisba.es (P.L.-A.); 3 Department of Surgery, Kangnam Sacred Heart Hospital, Hallym University, Seoul 07441, Republic of Korea; 4 Department of Pathology, Duke University, Durham, NC 27710, USA; rachel.factor@duke.edu; 5 Duke Cancer Institute, Durham, NC 27710, USA

**Keywords:** breast cancer, ER+/HER2−, epigenetics, lymph node metastasis, nodal burden, DNA methylation

## Abstract

De-escalation of axillary surgery has resulted in the loss of pathologic nodal information, yet the extent of lymph node involvement remains an important determinant of treatment decisions in estrogen receptor-positive (ER+)/HER2− disease. We examined whether primary tumors differed molecularly according to the extent of this regional dissemination. Genome-wide DNA methylation profiling of primary ER+/HER2− tumors from 47 patients with pN1 (*n* = 29) vs. >pN1 (*n* = 18) disease showed differences concentrated at promoters of developmental and cell-adhesion genes. By integrating methylomes with transcriptomes from the TCGA-BRCA cohort (*n* = 148) and clinical outcomes from KM Plotter (RFS, *n* = 1154; OS, *n* = 442; DMFS, *n* = 423), we identified four genes (*ARL10*, *RIC3*, *CXCL14*, *KCNH2*) showing concordant molecular and clinical associations, from which we derived the Lymph-node Involvement Outcome Numerator (LION) score. Lower LION scores were observed in metastatic lesions from the AURORA US cohort (*n* = 45). In SCAN-B (*n* = 3969), lower scores were associated with shorter distant recurrence-free intervals (HR = 0.38; 95% CI 0.23–0.62); this association persisted after adjustment for age, nodal and tumor category but was lost after adjustment for histological grade (HR = 0.83; 95% CI 0.48–1.44), indicating that the score and grade capture overlapping biology. These findings suggest that primary tumors already display coordinated epigenetic and transcriptional alterations associated with the extent of metastatic dissemination.

## 1. Introduction

Deeper insights into the biological heterogeneity of breast cancer (BC) and the availability of effective systemic therapies have fundamentally changed the surgical management of the axilla. Randomized trials have demonstrated that completion axillary lymph node dissection (ALND) does not improve locoregional control or overall survival in early-stage BC patients with minimal nodal disease identified at sentinel lymph node biopsy [1,2,3,4]. As a result, omission of ALND has become standard in selected patients, reducing the risk of lymphedema and functional morbidity without compromising oncologic outcomes [2].

In the neoadjuvant setting, high rates of nodal pathologic complete response (pCR) permit ALND omission in certain BC subtypes [5,6]. However, in patients with ER+/HER2− disease, rates rarely exceed 35% [7,8], and many of these patients still undergo ALND for residual nodal disease, an approach currently under evaluation in ongoing trials [9,10].

Collectively, these trials establish that surgical removal of axillary lymph nodes does not confer therapeutic benefit in selected patient populations, but they do not diminish the prognostic significance of the extent of nodal involvement. In ER+/HER2− disease, the extent of nodal metastases continues to govern systemic treatment decisions, including eligibility for genomic assays and CDK4/6 inhibitor-based therapies [11,12,13,14]. Axillary de-escalation therefore removes pathologic assessment of a variable that remains integral to treatment algorithms, increasingly requiring clinicians to make systemic decisions without granular nodal data. This raises the question of whether the extent of nodal involvement is associated with distinct molecular features already present within the primary tumor, potentially reflecting biological programs underlying metastatic dissemination. Among the different biological molecular layers, DNA methylation (DNAm) represents a promising readout because it integrates the cumulative effects of genetic, transcriptional, and environmental influences on tumor cell identity [15]. Aberrant DNAm contributes to transcriptional reprogramming, cellular plasticity, and metastatic competence [16], and can be measured in clinical tumor specimens.

Here, we used the extent of nodal involvement as a phenotypic measure of demonstrated regional metastatic behavior and assessed the extent to which this phenotype is reflected in the epigenetic landscape of the primary tumor. We performed genome-wide DNAm profiling of primary ER+/HER2− tumors from patients with limited (pN1) vs. extensive (>pN1) nodal disease and integrated the resulting alterations with independent DNAm, transcriptomic, and clinical datasets. We then summarized coordinated epigenetic alterations associated with nodal burden into a biologically informed molecular metric to examine their relationship with tumor biology and clinical outcomes. We hypothesized that these alterations reflect biological programs associated with nodal dissemination and progression.

## 2. Results

### 2.1. Clinicopathological Characteristics of the Study Cohort

A total of 51 female patients met the study inclusion criteria and were selected for methylation profiling. After DNA extraction and quality control, 47 cases yielded analyzable material and comprised the final study cohort (pN1, *n* = 29; >pN1, *n* = 18; Table 1 and Supplementary Table S1). Median age at diagnosis was 60 years (IQR: 39.5–80.5). Most tumors were pure invasive ductal carcinomas (74.5%), with the remainder showing mixed ductal and lobular histology (25.5%).

Overall, the two groups showed no significant differences in age (p = 0.45) or tumor histology (*p* = 0.74). As expected, higher nodal burden was associated with higher tumor category (*p* = 0.02) [17,18]. Most patients were White (68.1%), with fewer Black (17.0%) and other races (14.9%) included. While not statistically significant (*p* = 0.09), a slightly higher proportion of Black patients was observed in the >pN1 group. This study population formed the basis for subsequent analyses of epigenetic alterations associated with nodal burden.

### 2.2. Distinct DNA Methylation Patterns Characterize Breast Tumors with High Nodal Burden

Because DNAm profiles in bulk tumors can be influenced by non-tumor cell populations, we first assessed whether the two groups differed in tumor composition. Tumor purity, estimated using the LUMP method [19], was similar between pN1 and >pN1 tumors (*p* = 0.71; Table 1). Likewise, deconvolution of cell-type proportions revealed largely comparable immune and stromal landscapes across groups, with no significant differences and only a trend toward higher regulatory T-cell infiltration in >pN1 tumors (*p* = 0.078; Supplementary Figure S1). These analyses indicate that differences in tumor composition are unlikely to substantially confound the methylation comparisons between groups.

**Figure 1:**
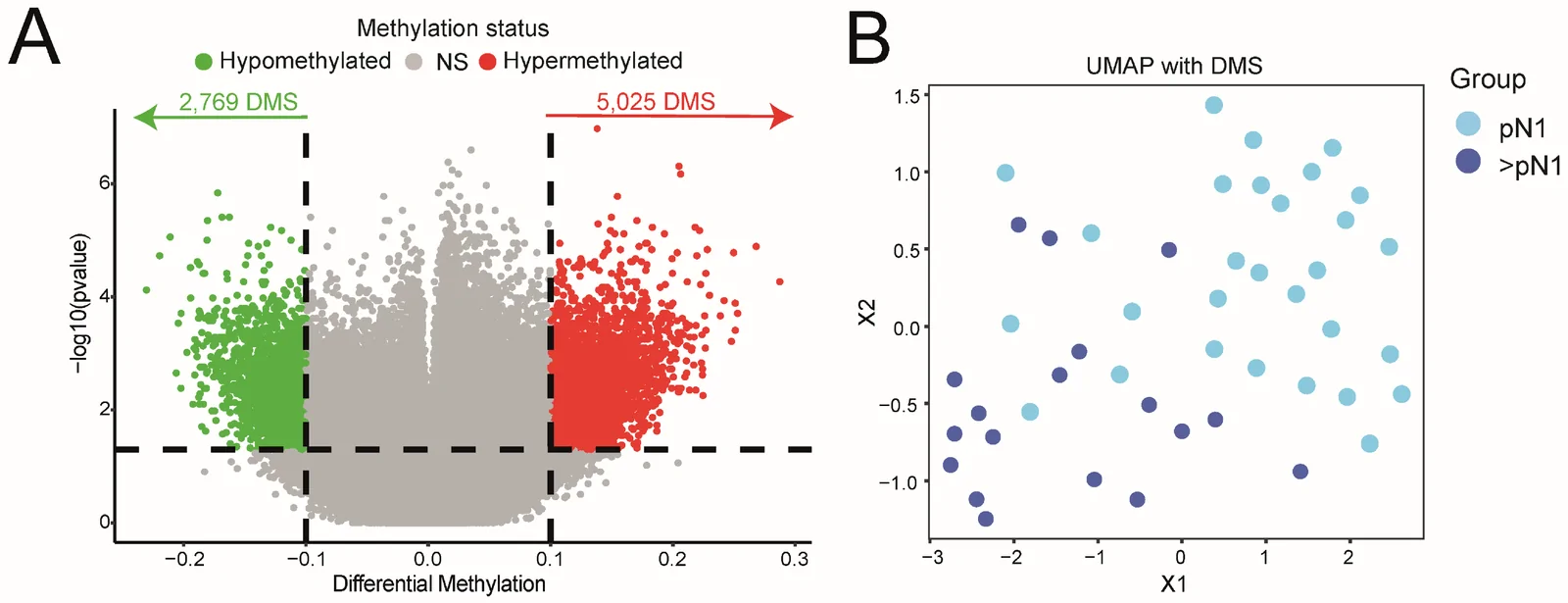
**Epigenetic landscape of differentially methylated CpG sites associated with lymph node involvement in primary breast tumors.** (**A**) Volcano plot of the DMS between pN1 and >pN1 tumors (*p* < 0.05; β-value >10%). 7794 DMS, of which 5025 were hypermethylated (red) and 2769 hypomethylated (green). (**B**) Uniform Manifold Approximation and Projection (UMAP) representation of the DNAm landscape of DMS, illustrating the distribution of DNAm profiles and their partial segregation according to nodal involvement. Dashed lines indicate the significance thresholds of |Δβ| = 0.10 (vertical lines) and *p* = 0.05 (horizontal line).

We then compared genome-wide DNAm profiles and identified 7794 differentially methylated CpG sites (DMS) between pN1 and >pN1 tumors (*p* < 0.05), with a predominance of hypermethylation (5025 DMS) over hypomethylation (2769 DMS) in >pN1 tumors (Figure 1A). These methylation patterns partially segregated patients according to nodal status (Figure 1B), despite some overlap between groups, indicating that primary tumors display epigenetic differences associated with the extent of lymph node involvement.

Tumors with higher nodal burden were characterized by promoter-associated hypermethylation alongside hypomethylation in non-promoter regions, a distribution consistent with the methylation patterns previously described in advanced disease [20] (Supplementary Figure S2A). Stratification by CpG island context revealed that hypermethylation was predominantly enriched within CpG islands, whereas hypomethylation was mainly distributed across open sea and shelf regions (Supplementary Figure S2B). For both hyper- and hypomethylated DMS, changes localized predominantly to promoter regions, followed by intragenic regions, with smaller contributions from enhancers, insulators, and distal intergenic sites. Detailed analyses of genomic distribution are provided in Supplementary Figure S2C,D.

Together, these findings indicate that primary ER+/HER2− breast tumors may carry coordinated DNAm traits associated with advanced nodal involvement.

### 2.3. Cross-Cohort Promoter Methylation in Higher-Nodal-Involvement Tumors Converges on Genes with Altered Expression

DNAm at promoters is one well-established mechanism of gene regulation and is frequently altered during tumor progression [21]. Given the enrichment of DNAm alterations in these regions, we focused on promoter-associated DMS in >pN1 vs. pN1 tumors.

**Figure 2:**
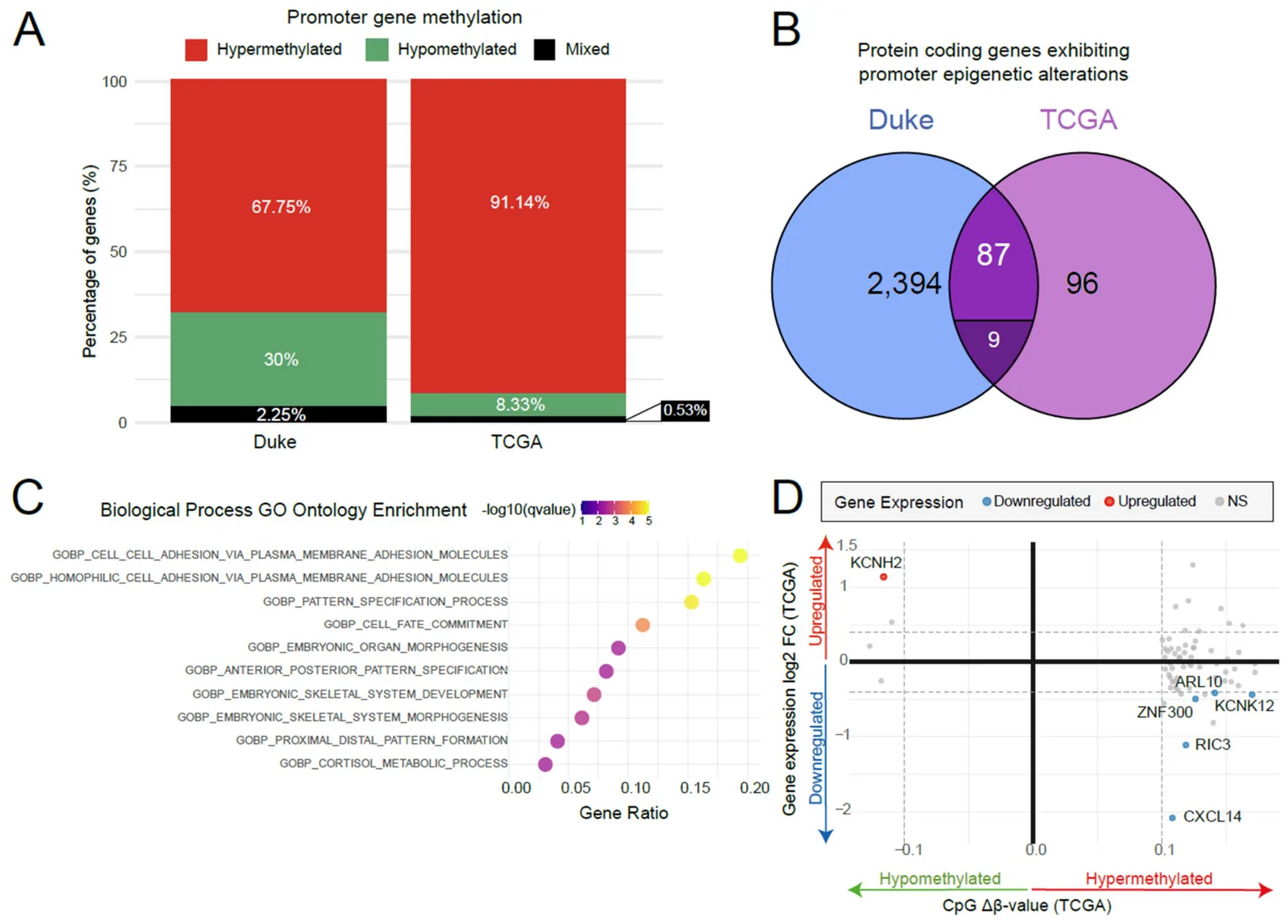
**Promoter DNA methylation and gene expression alterations associated with greater nodal involvement.** (**A**) Distribution of promoter-associated differentially methylated genes according to predominant methylation pattern in the Duke and TCGA cohorts. (**B**) Overlap of protein-coding genes showing promoter-associated methylation alterations in the Duke and TCGA cohorts. (**C**) Top 10 enriched Gene Ontology (GO) biological processes among genes with shared promoter-associated methylation alterations. (**D**) Integration of promoter methylation and gene expression identifies genes showing concordant methylation-expression alterations across cohorts. Genes located in the lower right quadrant (blue) are downregulated, and the genes in the upper left quadrant (red) are upregulated.

In the Duke cohort, 2490 protein-coding genes exhibited promoter-associated DNAm changes in tumors with higher nodal involvement, most of which (*n* = 1687) were characterized by promoter hypermethylation (Figure 2A). To evaluate the reproducibility of these findings, we analyzed 148 matched ER+/HER2− tumor samples from TCGA-BRCA (Supplementary Table S2, Supplementary Figure S3A), including DNAm and transcriptomic data from 108 pN1 and 40 >pN1 tumors. Their clinicopathological characteristics are summarized in Table 2.

Comparison of DNAm profiles identified epigenetic differences between nodal groups (*p* < 0.05; Supplementary Figure S3B), comprising 522 hypermethylated and 86 hypomethylated sites. Of the 3736 Duke DMSs represented on both platforms, effect sizes (Δβ) were moderately and significantly correlated with those observed in TCGA (Spearman ρ = 0.44, *p* < 0.001), indicating that the direction and relative magnitude of methylation differences were consistent across cohorts, despite the reduced number of sites reaching significance in TCGA. Similar to our in-house cohort, hypermethylated DMS in TCGA tended to localize in promoters (Supplementary Figure S3C) and CpG islands (Supplementary Figure S3D). Thus, we again focused on protein-coding genes with promoter-associated DMS. Hypermethylation remained the predominant alteration and was even more pronounced among the 192 differentially methylated genes identified in TCGA (91.1%; Figure 2A).

Across both cohorts, 96 genes showed promoter-associated methylation changes, 87 of which had a concordant epigenetic direction of change (Figure 2B). These genes were enriched in pathways related to development and cell adhesion, processes usually implicated in tumor progression (Figure 2C).

Integration with transcriptomic data from the TCGA cohort identified six genes with both differential promoter methylation and gene expression (*ARL10, CXCL14, KCNK12, KCNH2, RIC3, ZNF300*). Five genes showed promoter hypermethylation with reduced expression, whereas *KCNH2* presented hypomethylation along with increased expression (Figure 2D).

Since promoter DNAm is one of the several mechanisms that can shape gene expression, we examined copy-number alterations in the same TCGA patients. Alterations affecting these loci were infrequent and did not differ between nodal groups (Supplementary Figure S4). These findings suggest that copy-number alterations are unlikely to explain the observed expression differences, although they do not establish a causal contribution of promoter DNAm. Additional regulatory mechanisms were not evaluated in this study and therefore cannot be excluded. Gene-level methylation in the promoters and expression patterns are detailed in Supplementary Figure S5 and Supplementary Table S3.

### 2.4. Biological Characterization of the LION Score, a Four-Gene Readout of Dissemination-Associated Biology

To explore the biological relevance of these six genes, we examined their individual associations with clinical outcomes using the multi-institutional KM Plotter database, including relapse-free survival (RFS), overall survival (OS), and distant metastasis-free survival (DMFS; Supplementary Figure S6A). Associations were heterogeneous: *ZNF300* showed no significant association with any clinical parameter, and *KCNK12* was associated with RFS in the direction opposite to that expected from our methylation–expression analysis. Such variability at the single-gene level may reflect inter-patient differences and disease heterogeneity [22]. We therefore retained the four genes that were directionally consistent with our integrative methylation–expression findings and showed at least one significant clinical association (Supplementary Figure S6B). The stepwise prioritization strategy, including the selection criteria and number of genes retained at each stage, is summarized in Supplementary Table S7.

**Figure 3:**
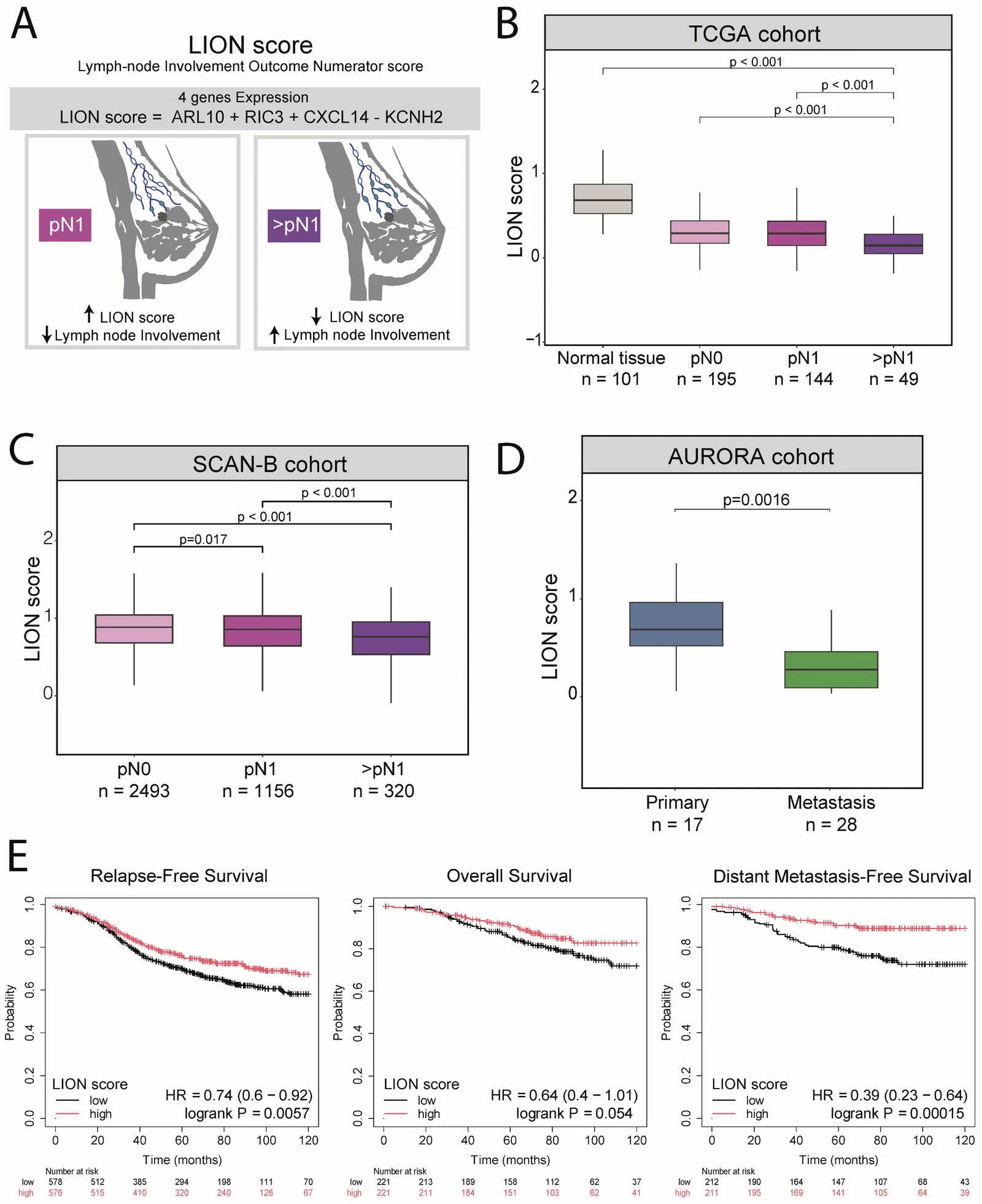
**LION score, a four-gene expression metric associated with lymph node involvement and metastatic progression in primary ER+/HER2− breast tumors.** (**A**) Schematic illustration of the LION score, integrating the expression of four genes. Arrows indicate the inverse association between the LION score and nodal involvement, with higher LION scores associated with lower nodal burden and lower LION scores with higher nodal burden. (**B**) LION score distribution in normal breast tissue and ER+/HER2− tumors stratified by nodal status in TCGA-BRCA. (**C**) Independent validation of the LION score according to nodal status in the SCAN-B cohort. (**D**) LION score distribution in primary and metastatic tumors from the AURORA US cohort. (**E**) Kaplan–Meier survival curves illustrating differences in RFS (**left**), OS (**middle**), and DMFS (**right**) in ER+/HER2− BC patients stratified by the LION score.

We reasoned that their coordinated behavior could provide a molecular summary of the alterations associated with regional dissemination. We therefore combined *KCNH2*, *ARL10*, *RIC3* and *CXCL14* into the Lymph-node Involvement Outcome Numerator (LION) score (more details in the Methods and Materials section; Figure 3A). Lower LION scores reflected a dissemination-associated molecular phenotype. The LION score should be interpreted as a descriptive summary of a coordinated methylation–expression pattern detectable in the primary tumor, rather than as a diagnostic or prognostic classifier.

### 2.5. The LION Program Tracks Histopathological Grade and Is Accentuated in Metastatic Disease

The LION score was further evaluated across several external datasets, summarized in Supplementary Table S4. In the TCGA cohort, the LION score was lower in tumors with >pN1 disease than in those with pN1 disease (median [IQR]: 0.144 [0.228] vs. 0.287 [0.288]; median difference = −0.143; *p* < 0.001), whereas pN0 and pN1 tumors showed comparable scores (Figure 3B). This pattern was reproduced in the independent SCAN-B cohort (*n* = 3969), where LION scores were lower in >pN1 than in pN1 tumors (median [IQR]: 0.760 [0.418] vs. 0.854 [0.388]; median difference = −0.094; *p* < 0.001; Figure 3C).

To assess whether this program extends to systemic dissemination, we evaluated the LION score in the AURORA US cohort [23] (*n* = 17 primary and *n* = 28 metastatic ER+/HER2− tumors). Scores were significantly lower in metastatic than in primary tumors (*p* = 0.0016; Figure 3D).

Consistent with these observations, associations between the LION score and clinical outcomes were examined in KM Plotter, with endpoint-specific sample sizes of 1154 for RFS, 442 for OS, and 423 for DMFS [24]. The sources of cases included in the analyses are detailed in Supplementary Table S5. Lower scores were associated with shorter DMFS (HR = 0.39; 95% CI: 0.23–0.64; *p* < 0.001; Figure 3E, right panel). The LION metric was also associated with shorter RFS (HR = 0.74; 95% CI: 0.60–0.92; *p* = 0.0057; Figure 3E, left panel), while the association with OS did not reach statistical significance (HR = 0.64; 95% CI: 0.40–1.01; *p* = 0.054; Figure 3E, middle panel).

**Figure 4:**
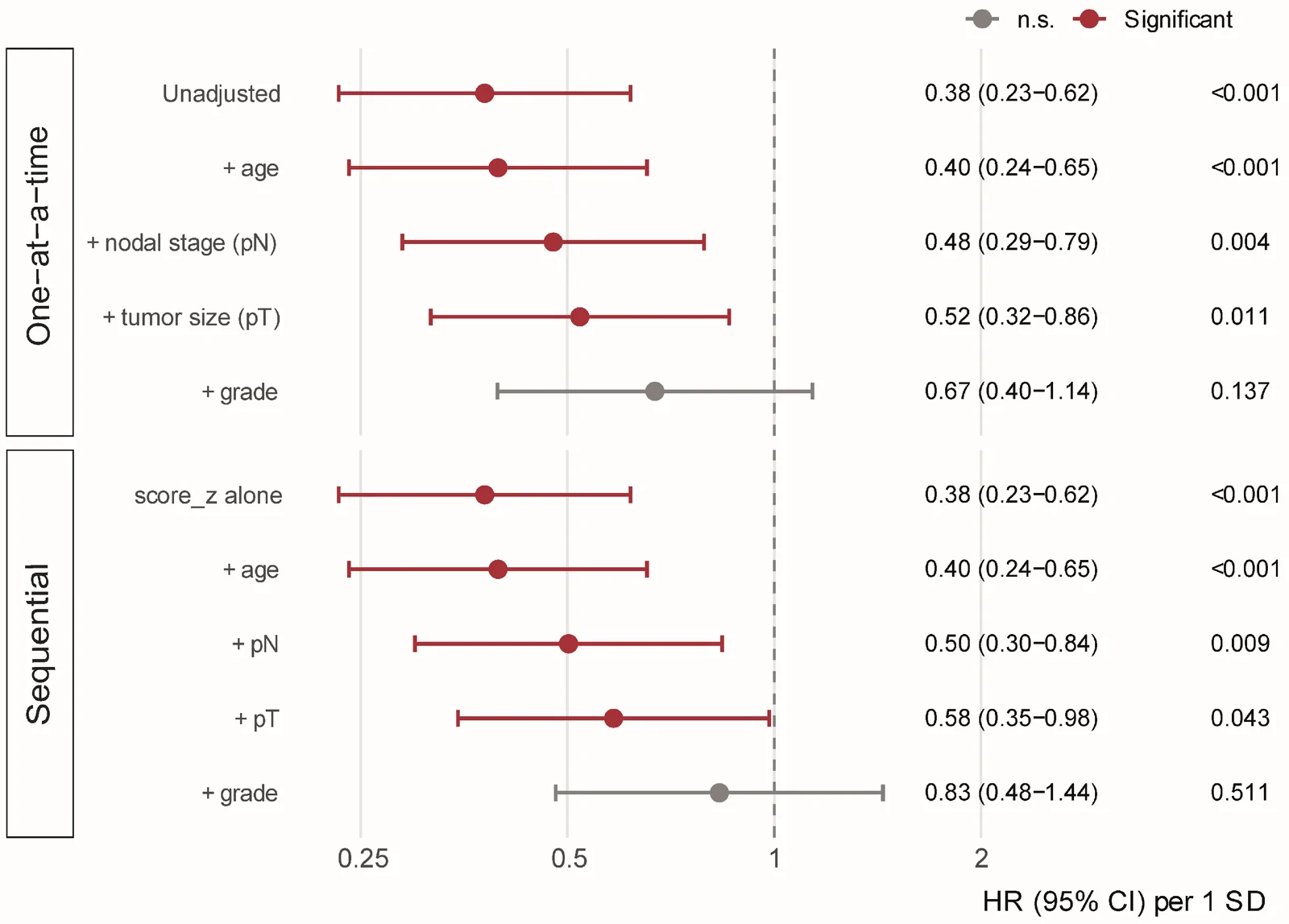
**Association of the LION Score with distant relapse-free interval across multivariable models.** Forest plots show hazard ratios (HRs) and 95% confidence intervals (CIs) for the LION Score, expressed per 1 standard deviation (SD), in the SCAN-B cohort. The association was evaluated using two modeling strategies: one-at-a-time adjustment, in which age, nodal stage (pN), tumor size (pT), and histological grade were added individually to the model, and sequential adjustment, in which covariates were added progressively. Red and gray estimates indicate statistically significant and non-significant associations, respectively. The vertical dashed line indicates the null value (HR = 1).

Because these analyses were univariable and were performed in the dataset used for gene selection, we next examined whether the association with distant recurrence persisted after accounting for established prognostic variables in an independent cohort. In SCAN-B, the LION score was associated with distant recurrence-free interval in univariable analysis (HR = 0.38; 95% CI: 0.23–0.62; *p* < 0.001). This association was progressively attenuated as covariates were added and was fully lost only after adjusting for histological grade (HR = 0.83; 95% CI: 0.48–1.44; *p* = 0.51; Figure 4), in which nodal stage (>pN1 vs. pN0: HR = 3.48; 95% CI: 2.30–5.28; *p* < 0.001), tumor category (T2 vs. T1: HR = 1.89; 95% CI: 1.32–2.69; *p* < 0.001) and grade (G3 vs. G1: HR = 3.10; 95% CI: 1.65–5.83; *p* < 0.001) remained independently associated with the endpoint, all included in Supplementary Table S6. Both sequential and one-at-a-time adjustment indicated that this attenuation was driven primarily by histological grade: the score remained significant after individual adjustment for age, nodal category, and tumor category (HR = 0.40–0.52; all *p* < 0.05), but was no longer significant when grade was included (HR = 0.67; 95% CI: 0.40–1.14; Figure 4).

Together, these findings indicate that primary ER+/HER2− tumors already display coordinated epigenetic and transcriptional alterations associated with the extent of nodal involvement, and that this program is further accentuated in metastatic disease, indicating that molecular features associated with regional and systemic dissemination are detectable in the primary tumors.

## 3. Discussion

The clinical management of nodal disease in BC is undergoing a fundamental shift. Axillary lymph node dissection, once standard for patients with nodal involvement, is increasingly omitted given the lack of demonstrated survival benefit and substantial risk of long-term morbidity. Yet in ER+/HER2− disease, the extent of nodal involvement continues to inform systemic treatment decisions, including the use of genomic assays and CDK4/6 inhibitors [11,12,13,14]. This evolving landscape provides an opportunity to investigate whether primary tumors harbor traceable molecular programs associated with regional dissemination.

In this study, we found that primary ER+/HER2− tumors with greater nodal involvement harbor coordinated epigenetic alterations accompanied by changes in gene expression. Promoter-associated methylation differences showed significant cross-cohort concordance, with a predominance of hypermethylation and enrichment of affected genes in developmental and cell-adhesion pathways. These findings support the existence of molecular programs associated with regional dissemination that are detectable in the primary tumor, rather than nodal involvement representing an anatomical manifestation of disease extent.

The biological processes identified are consistent with the acquisition of cellular plasticity and altered tumor–microenvironment interactions during tumor progression. Developmental and cell-adhesion programs are frequently disrupted or reactivated during cancer progression and can influence cellular identity, tissue organization, and metastatic competence [25]. Although the individual methylation changes identified here do not establish causality, their cross-cohort concordance and integration with transcriptional alterations support a coordinated molecular phenotype associated with greater nodal involvement.

To summarize these coordinated alterations, we developed the Lymph-node Involvement Outcome Numerator (LION) score, a gene expression-based metric from four genes identified through the integration of promoter methylation, gene expression, and clinical associations: *ARL10*, *RIC3*, *CXCL14*, and *KCNH2*. The score was used to summarize the coordinated behavior of these four genes. *CXCL14* has been linked to immune-cell infiltration, antigen presentation [26], and tumor progression [27]. Furthermore, *CXCL14* was incorporated into a metastasis-associated prognostic signature in BC, supporting its potential relevance in this process [28]. *ARL10*, involved in vesicle trafficking and cytoskeletal organization [29], and *RIC3*, implicated in inflammatory signaling [30], have both been reported to be downregulated in BC, potentially contributing to cellular plasticity and tumor–microenvironment remodeling associated with invasion [31,32]. *KCNH2*, the only gene in the score showing increased expression accompanied by promoter hypomethylation, has previously been associated with aggressive tumor behavior, metastatic potential, and lymph node involvement in BC [33,34]. Together, these observations suggest that the LION score captures coordinated alterations across biological processes relevant to tumor progression, while not implying a causal role for any individual component.

An important observation was that the dissemination-associated molecular phenotype was not restricted to regional disease. The LION score was lower in metastatic than in primary tumors in AURORA-US and was associated with shorter DMFS in KM Plotter, suggesting that the molecular features associated with nodal dissemination may extend to systemic metastatic progression. However, the prognostic association of LION was attenuated after adjustment for established clinicopathological variables, particularly histological grade. This indicates that the molecular information captured by LION substantially overlaps with histological grade and does not provide independent prognostic information beyond grade in the SCAN-B cohort. These findings therefore support interpreting LION primarily as a biologically informative molecular metric rather than as an independent prognostic tool.

Notably, LION was not developed as a clinical classifier of nodal stage. Unlike the EpiSig classifier we previously reported [35], the LION score was derived to summarize coordinated molecular alterations associated with a phenotype of regional dissemination. Accordingly, LION should be interpreted as a descriptive, biologically informed molecular metric rather than as a diagnostic, prognostic, or treatment-selection tool.

Overall, our findings suggest that primary ER+/HER2− tumors with greater regional lymphatic dissemination display coordinated epigenetic and transcriptional alterations that are detectable in the primary tumor. The LION score provides a compact molecular representation of this dissemination-associated biology. Rather than functioning as a replacement for nodal staging, it offers a framework for studying how tumor molecular states relate to regional and systemic dissemination. Future studies incorporating larger cohorts, longitudinal samples, and functional validation will be required to determine whether these programs can ultimately complement clinicopathological assessment or provide mechanistic insight into metastatic progression.

### Limitations

Several limitations should be considered. The sample size of our in-house cohort remains modest. In addition, the genome-wide discovery analysis relied on nominal significance combined with an effect-size threshold rather than formal FDR control, increasing the possibility of false-positive DMS in the discovery cohort. For this reason, individual DMS should be considered exploratory and prioritized only through subsequent cross-cohort analyses. The discovery cohort was, however, clinically homogeneous, with samples collected and processed at a single institution using the same EPIC DNAm platform, minimizing the biological and technical heterogeneity. Given differences in methylation array platforms, complete replication at the individual CpG level was not expected. Instead, our validation strategy focused on identifying promoter-associated methylation patterns and concordant gene expression. Nevertheless, the consistency of findings across two independent cohorts strengthens the validity of our conclusions.

Although this limited sample size of the discovery cohort precluded adequately powered multivariable analyses, we evaluated the LION score in the larger SCAN-B cohort, where its association with distant recurrence was significant in univariable analysis but was attenuated after adjustment for clinicopathological variables and was no longer significant after accounting for histological grade. A key limitation is that the prognostic utility of LION has not yet been formally validated in appropriately powered, independent cohorts. Therefore, we consider LION primarily as an informative readout of dissemination-associated tumor biology.

Importantly, the observed associations between promoter DNAm and gene expression do not establish a causal regulatory relationship. Copy-number alterations were assessed and did not appear to account for the observed differences in gene expression, although other regulatory mechanisms may contribute to the transcriptional changes observed. In addition, the biological functions of the four genes comprising the LION score were not experimentally validated in this study. Functional studies will therefore be required to determine whether these genes directly contribute to the molecular processes underlying regional and systemic dissemination.

Nodal burden can be influenced by stromal interactions, immune infiltration, and other factors beyond tumor-intrinsic biology. However, the comparable immune and stromal cell composition observed between nodal groups suggests that the differential DNAm patterns identified are unlikely to be driven primarily by differences in cellular composition.

Finally, the LION score was developed in a clinically homogeneous cohort of treatment-naïve patients with clinically node-positive ER+/HER2− invasive ductal carcinoma. Accordingly, the biological associations identified should be interpreted within this clinical context. Pure invasive lobular carcinomas were intentionally excluded from this pilot discovery study because of their distinct molecular characteristics and metastatic patterns [36]. Therefore, the applicability of the LION score to this histological subtype, as well as to patients receiving neoadjuvant systemic therapy, remains uncertain and warrants dedicated investigation in future studies. In addition, the demographic composition of our cohorts may limit the generalizability of these findings. Further validation in larger, prospectively collected cohorts with broader racial, ethnic, and demographic representation will be important to determine whether the biological associations identified here, and the LION score, are reproducible across diverse patient populations.

## 4. Materials and Methods

### 4.1. Inclusion and Ethics Statement

This study was conducted in accordance with the Declaration of Helsinki and was approved by the Duke Institutional Review Board (Pro00112216). Publicly available datasets were sourced from publicly available repositories and did not require IRB approval or patient-informed consent per the Code of Federal Regulations (45 CFR §46.104). All newly generated data were collected and analyzed in compliance with institutional and ethical guidelines.

### 4.2. Study Design and Independent Evaluation Strategy

This study was designed as a multi-step discovery, molecular integration, and external evaluation analysis (Supplementary Figure S7). Genome-wide DNAm profiling was initially performed in a clinically homogeneous Duke cohort of treatment-naïve ER+/HER2− BC patients, stratified according to pathological nodal burden (pN1 vs. >pN1). Concordant promoter-associated DNAm alterations were integrated with TCGA-BRCA transcriptomic data to identify candidate genes, which were subsequently evaluated for their association with survival intervals using the Kaplan–Meier Plotter (KM Plotter, https://kmplot.com, accessed on 23 July 2025). The resulting LION score was evaluated across external datasets for its association with nodal burden (TCGA-BRCA and SCAN-B) and metastatic status (AURORA US). KM Plotter was used for descriptive outcome analyses, whereas SCAN-B, which was not used in gene selection or score construction, provided an independent evaluation of the association with distant recurrence using multivariable Cox proportional hazards models adjusted for established clinicopathological variables.

### 4.3. Patient Selection and Inclusion Criteria of the Duke Cohort

We retrospectively identified a cohort of patients with ER+/HER2− BC and clinically positive lymph nodes at diagnosis who underwent ALND at Duke University between 2014 and 2023. Patients were stratified into two groups based on pathological nodal stage: pN1 (1–3 positive nodes) and >pN1 (4 or more positive nodes). Patients with clinically negative nodes who underwent sentinel lymph node biopsy, those who received NAC, or patients with pure invasive lobular carcinoma were excluded to minimize heterogeneity. Estrogen receptor positivity and HER2 negativity were defined according to ASCO/CAP guidelines [37].

### 4.4. Tissue Processing and DNA Isolation from Paraffin Tissue Sections

Homogeneous tumor sections (10 µm thick) from untreated surgical specimens of primary BC were microdissected using a needle to extract genomic DNA, minimizing contamination from peritumoral tissue. DNA was isolated using the Quick-DNA FFPE Kit (ZYMO Research, Cat. No: D3067, Irvine, CA, USA) following the manufacturer’s instructions. DNA concentration and purity were assessed by using a Qubit 3 Fluorometer (Q33216; Thermo Fisher Scientific, Carlsbad, CA, USA) with the Qubit™ dsDNA HS Assay Kit (Q32851, Invitrogen, Waltham, MA, USA).

### 4.5. Genome-Wide DNA Methylation Profiling and Data Processing

Genome-wide DNAm profiling was carried out at the Duke Molecular Genomics Core facility (Durham, NC, USA). DNAm was assessed using the Infinium Methylation EPIC v1.0 BeadChip (Illumina, Inc., San Diego, CA, USA) following the manufacturer’s protocol. Data normalization and quality control were conducted using the R package ChAMP (v.2.36.0) [38]. Raw intensity data were imported and further processed using default settings. Samples that failed to meet quality control criteria were excluded. Beta-Mixture Quantile (BMIQ) normalization was applied to normalize the array data, and the ComBat function was used to correct batch effects for array and slide features. Quality control showed 16,880 probes with a detection *p*-value above 0.01, which were therefore removed. The non-CG labeled probes (*n* = 2715) or probes containing single-nucleotide polymorphisms (*n* = 95,217) were removed from downstream analyses [39]. Additionally, 65 probes targeting chromosome Y were also discarded. β-values represent the ratio of the intensity of the methylated (M) bead types to the total intensity of both methylated and unmethylated (U) bead types at each CpG site. They can be calculated using the equation β = [M/(M + U)]. Tumor purity was assessed using the leukocyte unmethylation for purity (LUMP) method described by Aran et al. [19]. The obtained score ranges from 0 to 1, with values closer to 1 indicating higher tumor purity.

DMSs were identified as CpG sites with an absolute difference in mean β-value > 0.10 (|Δβ| > 0.10) and *p* < 0.05 between groups. The |Δβ| >0.10 threshold was used as an effect-size filter to prioritize CpG sites showing larger methylation differences for subsequent cross-cohort evaluation. No formal genome-wide multiple-testing correction was applied at the discovery stage; therefore, these DMSs were considered exploratory candidates for subsequent cross-cohort prioritization rather than genome-wide significant loci. The same effect-size and nominal significance criteria were applied in TCGA for cross-cohort evaluation.

### 4.6. Cellular Deconvolution Based on DNA Methylation Profiling

Estimated cell subpopulation proportions were derived from normalized DNAm β-values using the methylCIBERSORT (v.0.2.0) R package and the breast_v2_Signature as the signature matrix [40]. Analysis parameters included disabling quantile normalization and 1000 permutations per run.

### 4.7. Characterization of Gene Regulatory Elements

Enhancer elements annotations were obtained from the FANTOM5 database [41]. CTCF motifs identified using the genome-wide position weight matrix scanner (PWMScan, https://epd.expasy.org/pwmtools/pwmscan.php?utm_source=chatgpt.com, accessed on 14 April 2025) of the JASPAR (https://jaspar.elixir.no/?utm_source=chatgpt.com (accessed on 14 April 2025)) CORE 2020 vertebrate motif library [42] were classified as Insulator Elements (IE). Genomic region annotations were retrieved using the *annotatePeak* function from ChIPseeker (Galaxy Version 1.28.3) [43], categorizing regions into promoter (≤1 kb, 1–2 kb, 2–3 kb), 5’ UTR, 3’ UTR, Exon, Intron, Downstream, and Intergenic. Accordingly, the distribution of genomic regions was analyzed, considering the following categories: (i) Distal intergenic; (ii) Intragenic (Exon, Intron, 5’ UTR, 3’ UTR, Downstream); (iii) Promoter (Promoter ≤ 1 kb, Promoter 1–2 kb, Promoter 2–3 kb); (iv) Insulators; (v) Enhancers. BED files were processed using Bedtools (v2.30.0) [44] within the Galaxy open-source platform [45].

### 4.8. Public Data Access and Processing

To evaluate the reproducibility of our findings, clinical and molecular data from the TCGA-BRCA project were obtained via the National Cancer Institute’s Genomic Data Commons portal using the TCGAbiolinks R package (v2.28.4) [46]. Only patients with ER+/HER2− tumors, identified by immunohistochemistry subtyping, were included. Patients with positive lymph nodes and either Invasive Ductal Carcinoma or Mixed Ductal and Lobular Carcinoma were selected to match the Duke cohort. Patients were included if they had both DNAm data (Illumina HumanMethylation450 array) and RNA sequencing (RNA-seq) data available for integrative molecular analysis. Patients who received NAC were excluded.

### 4.9. Copy-Number Alteration Analysis

Copy-number alteration data for the TCGA-BRCA cohort were obtained from genotyping array data generated using the Affymetrix Genome-Wide Human SNP 6.0 Array (Affymetrix, Santa Clara, CA, USA). Copy-number states were evaluated for the six genes (*ARL10*, *CXCL14*, *KCNH2*, *KCNK12*, *RIC3*, and *ZNF300*) in patients with pN1 and >pN1 disease (*n* = 148). Copy-number states were categorized as gain (copy number > 2), diploid (copy number = 2), or loss (copy number = 1).

### 4.10. Lymph-Node Involvement Outcome Numerator Score Calculation and External Evaluation

Genes with consistent differential methylation across datasets and concordant changes in gene expression were identified. The Lymph-node Involvement Outcome Numerator (LION) score was constructed by normalizing the raw expression counts of selected genes using size factors with the DESeq2 R package (v1.42.1) [47], and then scaling the normalized values with the minMax function from the Biobase (v2.62.0) R package. The LION score was then obtained by summing the individual scores of the three genes (*ARL10, RIC3* and *CXCL14*) that were hypermethylated at their promoter regions and downregulated in >pN1 tumors, and subtracting the individual score of *KCNH2*, the only gene found to be hypomethylated and upregulated in the same group.

We analyzed the robustness of the LION score distribution across different gene expression datasets of ER+/HER2− BC patients (Supplementary Table S4). Specifically, in the TCGA-BRCA cohort, we compared normal breast tissue with BC samples stratified by nodal status (pN0, pN1, >pN1). For these LION score distribution analyses, we used all ER+/HER2− TCGA-BRCA samples with available expression and nodal annotations; this cohort was therefore larger than the paired DNAm–RNA-seq subset (*n* = 148) used for the integrative molecular analysis. Furthermore, clinical and gene expression data from the SCAN-B cohort [48] were downloaded from the Mendeley repository (DOI: 10.17632/yzxtxn4nmd.4). Data from the AURORA US Metastasis cohort were retrieved from the NCBI GEO (GSE209998, https://www.ncbi.nlm.nih.gov/geo/, accessed on 6 June 2025) [23].

Survival analyses were performed using the gene expression data from the GeneChip platform available in the Kaplan–Meier plotter database [24]. Patients were restricted to ER+/HER2− tumors according to the corresponding microarray-based annotations. The LION score was implemented as a custom four-gene metric using the Affymetrix probe sets *ARL10* (1553207_at), *RIC3* (220282_at), *KCNH2* (205262_at), and *CXCL14* (222484_s_at). The score was calculated using the mean expression of the selected genes, with *KCNH2* included as an inverted component according to the LION score definition. Patients were dichotomized using the median LION score, and survival analyses were performed using a follow-up threshold of 120 months. Because each survival endpoint within the KM Plotter database includes only patients fulfilling the selected molecular criteria and with complete follow-up information, the number of patients differed between RFS (*n* = 1154), OS (*n* = 442), and DMFS (*n* = 423) analyses. The contribution of each dataset to the different survival endpoints is summarized in Supplementary Table S5.

### 4.11. Survival Modeling and Multivariable Analysis

Time-to-event analyses in the SCAN-B cohort used distant recurrence-free interval (DRFi) as the primary endpoint, with recurrence-free interval (RFi) and OS as secondary endpoints. Follow-up time was computed in years, and Cox proportional hazards models were fitted using the survival R package (v3.5-8). The LION score was modeled as a continuous variable scaled to units of one standard deviation (score_z), so that hazard ratios (HRs) are interpretable per 1-SD increment. Covariates comprised nodal stage (N0, N1, N23), tumor category (T1, T2, T3–T4), histological grade (NHG 1–3) and age at diagnosis. Multivariable models were restricted to complete cases across all included covariates, and the corresponding number of patients and events is reported for each model.

To evaluate whether the association between the LION score and distant recurrence was independent of established prognostic variables, we first estimated the univariable HR for score_z and then compared it with a fully adjusted model. To identify which covariate accounted for the attenuation of the score, we performed a sequential adjustment analysis in which each variable was added cumulatively to the model, as well as a complementary analysis in which score_z was adjusted for each covariate individually. In every model, the HR, 95% CI, and *p*-value of score_z were extracted using the broom R package (v1.0.5) and summarized in forest plots displaying the HRs and corresponding 95% CIs across adjustment models. The proportional hazards assumption was assessed using scaled Schoenfeld residuals (cox.zph function), and all HRs are reported with two-sided *p*-values.

### 4.12. Data Processing, Bioinformatic Analyses, and Visualization

Data processing and visualization were performed using multiple R packages. The Uniform Manifold Approximation and Projection for Dimension Reduction (UMAP) technique was used to visualize the distribution of groups according to their DMS using the M3C R package (v1.24.0). Forest plots were generated using the forestploter (v1.1.3) R package. Heatmaps were generated using the pheatmap (v1.0.12) R package. RNA-seq data from TCGA were analyzed using the DESeq2 R package (v1.42.1) [47]. Genes with a base mean of 25 reads or fewer were excluded. Differentially expressed genes were defined by an absolute log_2_ fold change (FC) >0.4 and a *p*-value < 0.05. Gene Ontology (GO) enrichment analysis was performed using the clusterProfiler (v4.10.1) R package [49]. Kaplan–Meier curves were generated using the Kaplan–Meier plotter tool [24], and the log-rank test was applied to assess the statistical significance of differences in survival rates. Data transformation was carried out using the tidyverse (v2.0.0) R package. For data manipulation and visualization, the ggplot2 (v3.4.4) and Upset (v1.4.0) R packages were used.

### 4.13. Statistics and Reproducibility

All statistical analyses were performed using R software (v.4.3.1). Categorical variables were analyzed with Fisher’s exact test. Continuous variables were assessed for normality using the Shapiro–Wilk test; variables following a normal distribution were compared using the two-sided Student’s *t*-test, whereas non-normally distributed variables were analyzed using the two-sided Wilcoxon rank-sum test.

Statistical differences between pN1 and >pN1 at each CpG site in methylation data were assessed using the two-sided Wilcoxon rank-sum test. Odds ratios (ORs) with 95% confidence intervals (CIs) were calculated using the questionR (v.0.7.8) R package with derived *p*-values obtained using Fisher’s exact test. The log-rank test was applied to assess the statistical significance of differences in survival rates.

## 5. Conclusions

In this study, we identified promoter-associated DNAm alterations showing significant cross-cohort concordance in primary ER+/HER2− breast tumors with greater nodal involvement, including coordinated changes in gene expression across independent cohorts. These alterations were enriched in genes involved in developmental and cell-adhesion processes, supporting the presence of tumor-associated molecular programs linked to regional lymphatic dissemination. Integration of these findings identified four genes: *ARL10*, *RIC3*, *CXCL14*, and *KCNH2*, that were combined into the LION score as a molecular readout of this dissemination-associated biology. LION was lower in tumors with greater nodal involvement and in metastatic tumors and was associated with distant recurrence. However, this association was attenuated after adjustment for histological grade. Thus, LION should currently be considered a biologically informative metric rather than a clinical classifier or validated prognostic tool. Further functional and prospective studies are warranted to determine the biological and clinical relevance of the molecular programs captured by LION.

## Figures and Tables

**Table 1:** Demographic and clinicopathologic features of the Duke cohort according to pN category.

	All Patients (*n* = 47)	ER+ HER2− pN1 (*n* = 29)	ER+ HER2− >pN1 (*n* = 18)
**Age (Years)**			*p* = 0.45 (a)
Median (IQR)	60 (39.5–80.5)	57 (37.0–77.0)	60.5 (52.5–71)
Mean (SD)	58.7 (14.2)	57.5 (14.7)	60.72 (13.42)
**Race**			*p* = 0.09 (b)
White	32 (68.1%)	22 (75.9%)	10 (55.55%)
Black	8 (17.0%)	2 (6.9%)	6 (33.33%)
Others	7 (14.9%)	5 (17.2%)	2 (11.11%)
**Tumor Histology**			*p* = 0.74 (b)
Ductal	35 (74.5%)	21 (75.9%)	14 (77.78%)
Mixed	12 (25.5%)	8 (24.1%)	4 (22.22%)
**cT category**			*p* = 0.02 (b)
T1	8 (17.0%)	7 (24.1%)	1 (5.56%)
T2	27 (57.5%)	18 (62.1%)	9 (50.00%)
T3	11 (23.4%)	3 (10.3%)	8 (44.44%)
T4	1 (2.1%)	1 (3.5%)	0 (0%)
**LUMP purity**			*p* = 0.71 (a)
Median (IQR)	0.63 (0.48–0.78)	0.64 (0.47–0.81)	0.62 (0.53–0.71)
Mean (SD)	0.65 (0.11)	0.65 (0.11)	0.64 (0.10)

(a) Student’s *t*-test; (b) Fisher’s exact test.

**Table 2:** Demographic and clinicopathologic features of the TCGA ER+/HER2− BC cohort according to pN category.

	All Patients (*n* = 148)	ER+ HER2− pN1 (*n* = 108)	ER+ HER2− >pN1 (*n* = 40)
**Age (Years)**			*p* = 0.706 (a)
Median (IQR)	55.0 (39.0–71.0)	56 (47–63)	53.5 (46.5–64.25)
Mean (SD)	55.3 (13.0)	55.06 (12.33)	56.05 (14.79)
**Race**			*p* = 0.456 (b)
White	110 (74.3%)	83 (76.85%)	27 (67.5%)
Black	31 (21.0%)	20 (18.52%)	11 (27.5%)
Others	7 (4.7%)	5 (4.63%)	2 (5%)
**Tumor Histology**			*p* = 0.612 (b)
Ductal	143 (96.6%)	105 (97.22%)	38 (95%)
Mixed	5 (3.4%)	3 (2.78%)	2 (5%)
**cT category**			*p* = 0.045 (b)
T1	37 (25.0%)	32 (29.63%)	5 (12.5%)
T2	95 (64.2%)	67 (62.04%)	28 (70%)
T3	16 (10.8%)	9 (8.33%)	7 (17.5%)
**LUMP purity**			*p* = 0.92 (c)
Median (IQR)	0.85 (0.69–1.00)	0.85 (0.70–1.00)	0.83 (0.67–1.00)
Mean (SD)	0.82 (0.12)	0.82 (0.12)	0.82 (0.12)

(a) Student’s *t*-test; (b) Fisher’s exact test; (c) Wilcoxon-rank sum test.

## Data Availability

The newly generated DNA methylation data from the Duke cohort are deposited in NCBI GEO under accession number GSE307770. TCGA methylation and gene expression data can be accessed from the National Cancer Institute GDC Data Portal (https://portal.gdc.cancer.gov/, accessed on 18 March 2025). Gene expression data from the SCAN-B cohort are available from the Mendeley repository (DOI: 10.17632/yzxtxn4nmd.4). Data from the AURORA US Metastasis cohort were retrieved from NCBI GEO (GSE209998). No specific code was generated for this project. All R scripts were adapted from the standard scripts provided in the packages described in the Materials and Methods section.

## Supplementary Materials

The following supporting information can be downloaded at: https://www.mdpi.com/article/10.3390/ijms27177901/s1.

## Abbreviations

The following abbreviations are used in this manuscript:

ALND	Axillary Lymph Node Dissection
BC	Breast Cancer
BMIQ	Beta-Mixture Quantile
CDK4/6	Cyclin-Dependent Kinase 4 and 6
CI	Confidence Interval
DEGs	Differentially Expressed Genes
DMFS	Distant Metastasis-Free Survival
DMS	Differentially Methylated CpG Sites
DNAm	DNA Methylation
DRFi	Distant Recurrence-Free interval
EPIC	Infinium MethylationEPIC BeadChip
EE	Enhancer Elements
ER+	Estrogen Receptor-Positive
FC	Fold Change
FFPE	Formalin-Fixed Paraffin-Embedded
GEO	Gene Expression Omnibus
GO	Gene Ontology
HER2−	Human Epidermal Growth Factor Receptor 2-negative
HR	Hazard Ratio
IE	Insulator Elements
IQR	Interquartile Range
IRB	Institutional Review Board
KM	Kaplan–Meier
LION	Lymph-node Involvement Outcome Numerator
LUMP	Leukocytes Unmethylation for Purity
NAC	Neoadjuvant Chemotherapy
OR	Odds Ratio
OS	Overall Survival
pCR	Pathologic Complete Response
pN1	Pathological Nodal Stage 1
>pN1	Pathological Nodal Stage with ≥4 Positive Lymph Nodes
pT	Pathological Tumor Stage
RFi	Relapse-Free interval
RFS	Relapse-Free Survival
RNA-seq	RNA Sequencing
SCAN-B	Sweden Cancerome Analysis Network—Breast
SD	Standard Deviation
TCGA	The Cancer Genome Atlas
TSS	Transcription Start Site
UMAP	Uniform Manifold Approximation and Projection
UTR	Untranslated Region
