# Supplementary material for: Primary Tumor Epigenetic and Transcriptomic Alterations Associated with Nodal Burden and Metastatic Risk in ER+/HER2− Breast Cancer
Source: Int J Mol Sci. 2026 Sep 4;27(17):7901. doi: 10.3390/ijms27177901 (PMC13566309; doi:10.3390/ijms27177901)
Supplement: Supplementary file 1 [file ijms-27-07901-s001.zip › Supplementary Figures and legends.pdf]

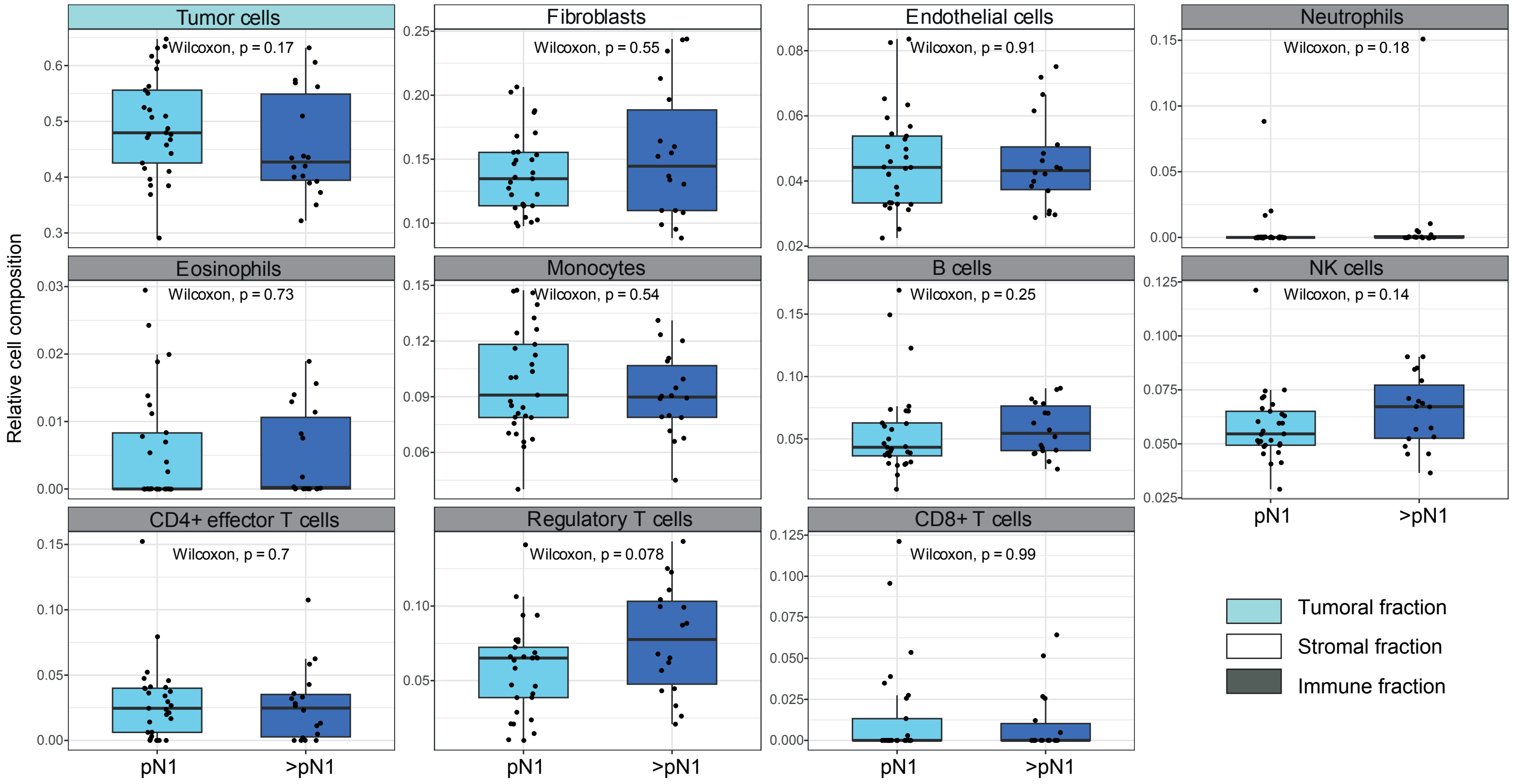

Relative cell composition
Tumor cells
Wilcoxon, p = 0.17
Fibroblasts
Wilcoxon, p = 0.55
Endothelial cells
Wilcoxon, p = 0.91
Neutrophils
Wilcoxon, p = 0.18
Eosinophils
Wilcoxon, p = 0.73
Monocytes
Wilcoxon, p = 0.54
B cells
Wilcoxon, p = 0.25
NK cells
Wilcoxon, p = 0.14
CD4+ effector T cells
Wilcoxon, p = 0.7
Regulatory T cells
Wilcoxon, p = 0.078
CD8+ T cells
Wilcoxon, p = 0.99
pN1
>pN1
Tumoral fraction
Stromal fraction
Immune fraction

Supplementary Figure S1. Cellular deconvolution of pN1 and >pN1 BC primary tumors of the Duke cohort using DNAm. Boxplots representing no significant differences in the composition of tumor microenvironment cells. It includes tumoral, stromal, and immune fractions. pN1 is shown in light blue and >pN1 in dark blue. The Wilcoxon rank-sum test was used to test statistical differences.

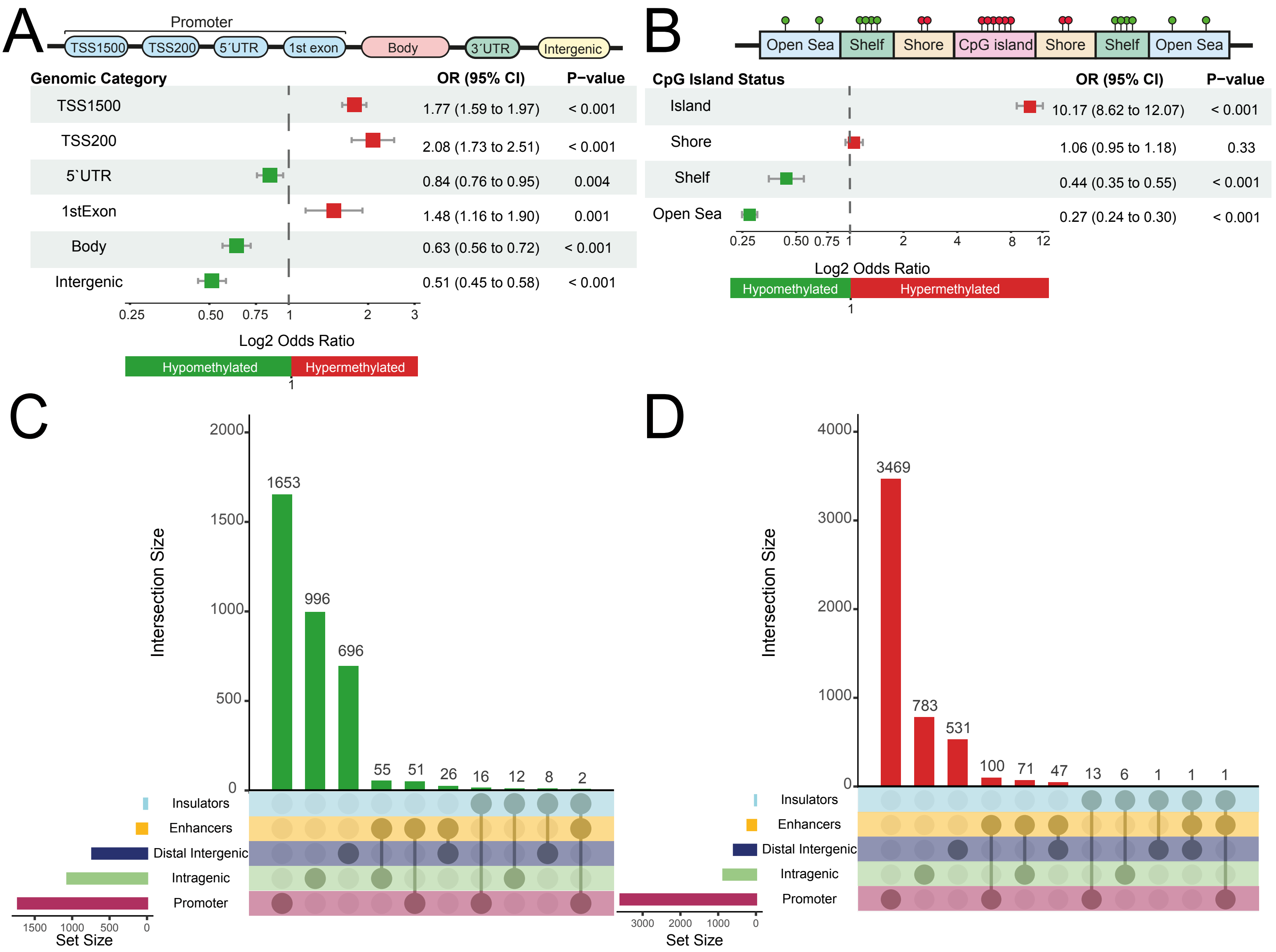
A
Promoter
TSS1500
TSS200
5´UTR
1st exon
Body
3´UTR
Intergenic
Genomic Category
OR (95% CI)
P−value
TSS1500 1.77 (1.59 to 1.97) < 0.001
TSS200 2.08 (1.73 to 2.51) < 0.001
5`UTR 0.84 (0.76 to 0.95) 0.004
1stExon 1.48 (1.16 to 1.90) 0.001
Body 0.63 (0.56 to 0.72) < 0.001
Intergenic 0.51 (0.45 to 0.58) < 0.001
0.25 0.50 0.75 1 2 3
Log2 Odds Ratio
Hypomethylated
Hypermethylated
1
B
Open Sea
Shelf
Shore
CpG island
Shore
Shelf
Open Sea
CpG Island Status
OR (95% CI)
P−value
Island 10.17 (8.62 to 12.07) < 0.001
Shore 1.06 (0.95 to 1.18) 0.33
Shelf 0.44 (0.35 to 0.55) < 0.001
Open Sea 0.27 (0.24 to 0.30) < 0.001
0.25 0.50 0.75 1 2 4 8 12
Log2 Odds Ratio
Hypomethylated
Hypermethylated
1
C
Intersection Size
2000
1500
1000
500
0
1653
996
696
55
51
26
16
12
8
2
Insulators
Enhancers
Distal Intergenic
Intragenic
Promoter
1500 1000 500 0
Set Size
D
Intersection Size
4000
3000
2000
1000
0
3469
783
531
100
71
47
13
6
1
1
1
Insulators
Enhancers
Distal Intergenic
Intragenic
Promoter
3000 2000 1000 0
Set Size

Supplementary Figure S2. Distribution of differentially methylated sites across genomic features and regulatory elements. (A) Odds ratio showing the distribution of the DMS across distinct genomic regions. **(B)** Odds ratio showing the distribution of the DMS according to their CpG category. Red denotes categories enriched in hypermethylated DMS, whereas green denotes enrichment of hypomethylated DMS. **(C)** UpSet plot showing the distribution of hypomethylated DMS across major gene regulatory elements. **(D)** UpSet plot illustrating the overlap of hypermethylated DMS among key gene regulatory elements.

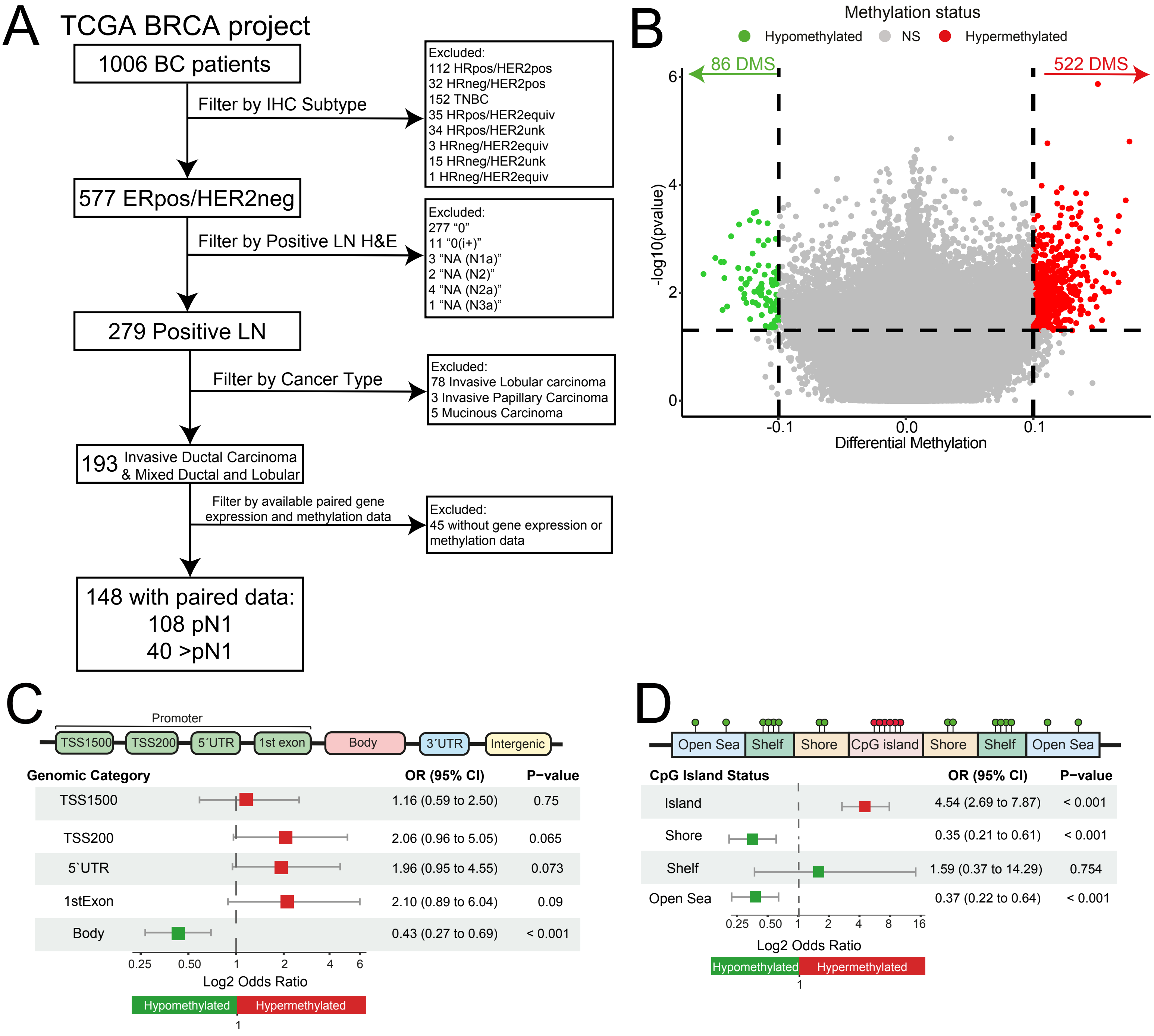
A
TCGA BRCA project
1006 BC patients
Filter by IHC Subtype
Excluded:
112 HRpos/HER2pos
32 HRneg/HER2pos
152 TNBC
35 HRpos/HER2equiv
34 HRpos/HER2unk
3 HRneg/HER2equiv
15 HRneg/HER2unk
1 HRneg/HER2equiv
577 ERpos/HER2neg
Filter by Positive LN H&E
Excluded:
277 "0"
11 "0(i+)"
3 "NA (N1a)"
2 "NA (N2)"
4 "NA (N2a)"
1 "NA (N3a)"
279 Positive LN
Filter by Cancer Type
Excluded:
78 Invasive Lobular carcinoma
3 Invasive Papillary Carcinoma
5 Mucinous Carcinoma
193 Invasive Ductal Carcinoma & Mixed Ductal and Lobular
Filter by available paired gene expression and methylation data
Excluded:
45 without gene expression or methylation data
148 with paired data:
108 pN1
40 >pN1
B
Methylation status
Hypomethylated
NS
Hypermethylated
86 DMS
522 DMS
-log10(pvalue)
Differential Methylation
-0.1
0.0
0.1
C
Promoter
TSS1500
TSS200
5´UTR
1st exon
Body
3´UTR
Intergenic
Genomic Category
OR (95% CI)
P−value
TSS1500
1.16 (0.59 to 2.50)
0.75
TSS200
2.06 (0.96 to 5.05)
0.065
5`UTR
1.96 (0.95 to 4.55)
0.073
1stExon
2.10 (0.89 to 6.04)
0.09
Body
0.43 (0.27 to 0.69)
< 0.001
Log2 Odds Ratio
Hypomethylated
Hypermethylated
D
Open Sea
Shelf
Shore
CpG island
Shore
Shelf
Open Sea
CpG Island Status
OR (95% CI)
P−value
Island
4.54 (2.69 to 7.87)
< 0.001
Shore
0.35 (0.21 to 0.61)
< 0.001
Shelf
1.59 (0.37 to 14.29)
0.754
Open Sea
0.37 (0.22 to 0.64)
< 0.001
Log2 Odds Ratio
Hypomethylated
Hypermethylated

Supplementary Figure S3. Validation cohort from the TCGA-BRCA dataset of ER +/HER2- BC patients stratified by lymph node involvement category. (A) Schematic overview of the patient inclusion workflow from the TCGA-BRCA dataset, highlighting clinical and molecular filtering steps. **(B)** Volcano plot of the 608 DMS between pN1 and >pN1 tumors ($p<0.05$; β-value >10%), of which 522 were hypermethylated (red) and 86 hypomethylated (green). **(C)** Odds ratio showing the distribution of the DMS across distinct genomic regions. **(D)** Odds ratio showing the distribution of the DMS according to their CpG category. Red denotes categories enriched in hypermethylated DMS, whereas green denotes enrichment of hypomethylated DMS.

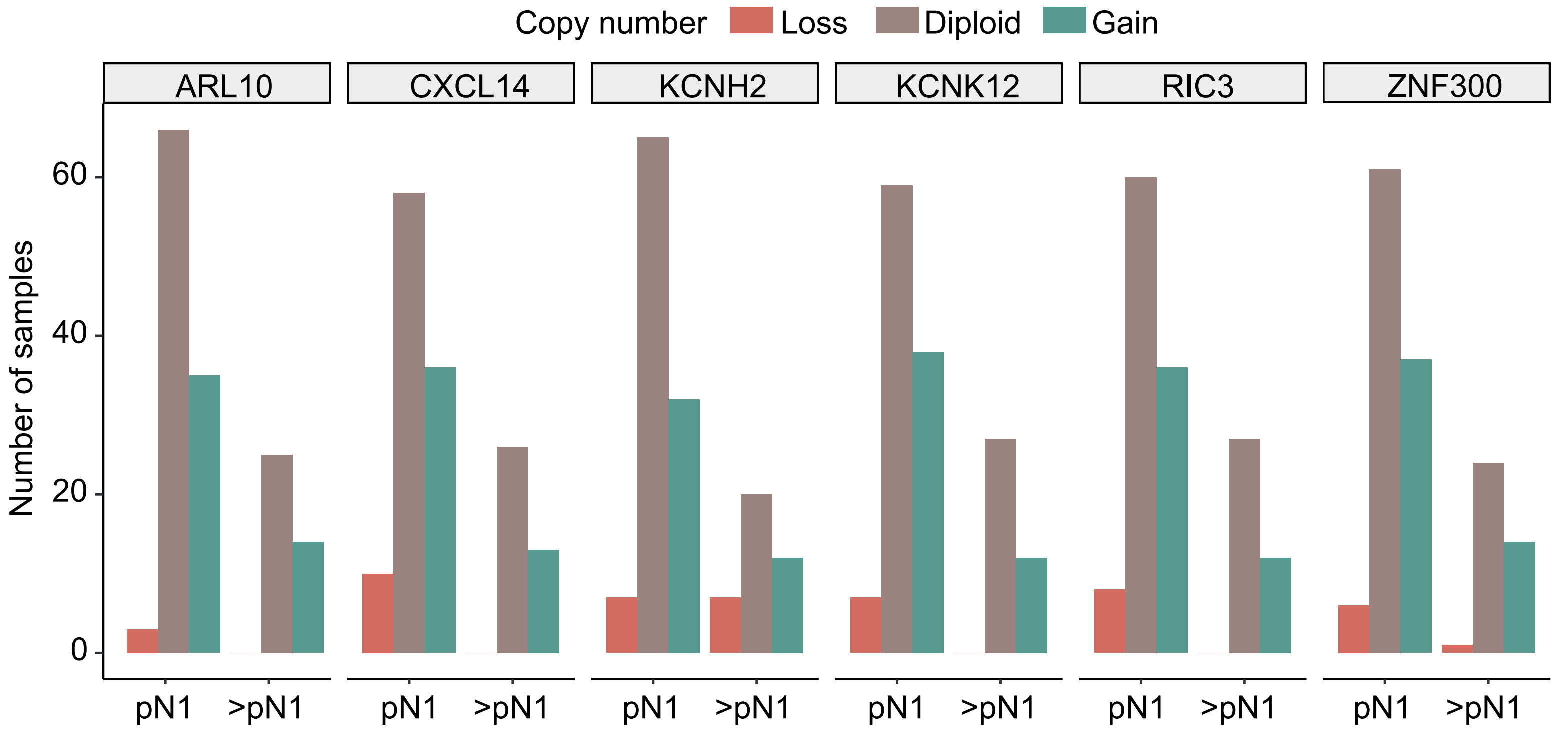

Copy number
Loss
Diploid
Gain
ARL10
CXCL14
KCNH2
KCNK12
RIC3
ZNF300
Number of samples
0
20
40
60
pN1
>pN1

Supplementary Figure S4. Copy-number variation profiles of genes exhibiting concordant methylation and expression patterns across nodal stages in the TCGA cohort. Stacked bar plots show the distribution of copy number states, loss, diploid, and gain, for *ARL10*, *CXCL14*, *KCNH2*, *KCNK12*, *RIC3*, and *ZNF300* in tumors with pN1 and >pN1 disease.

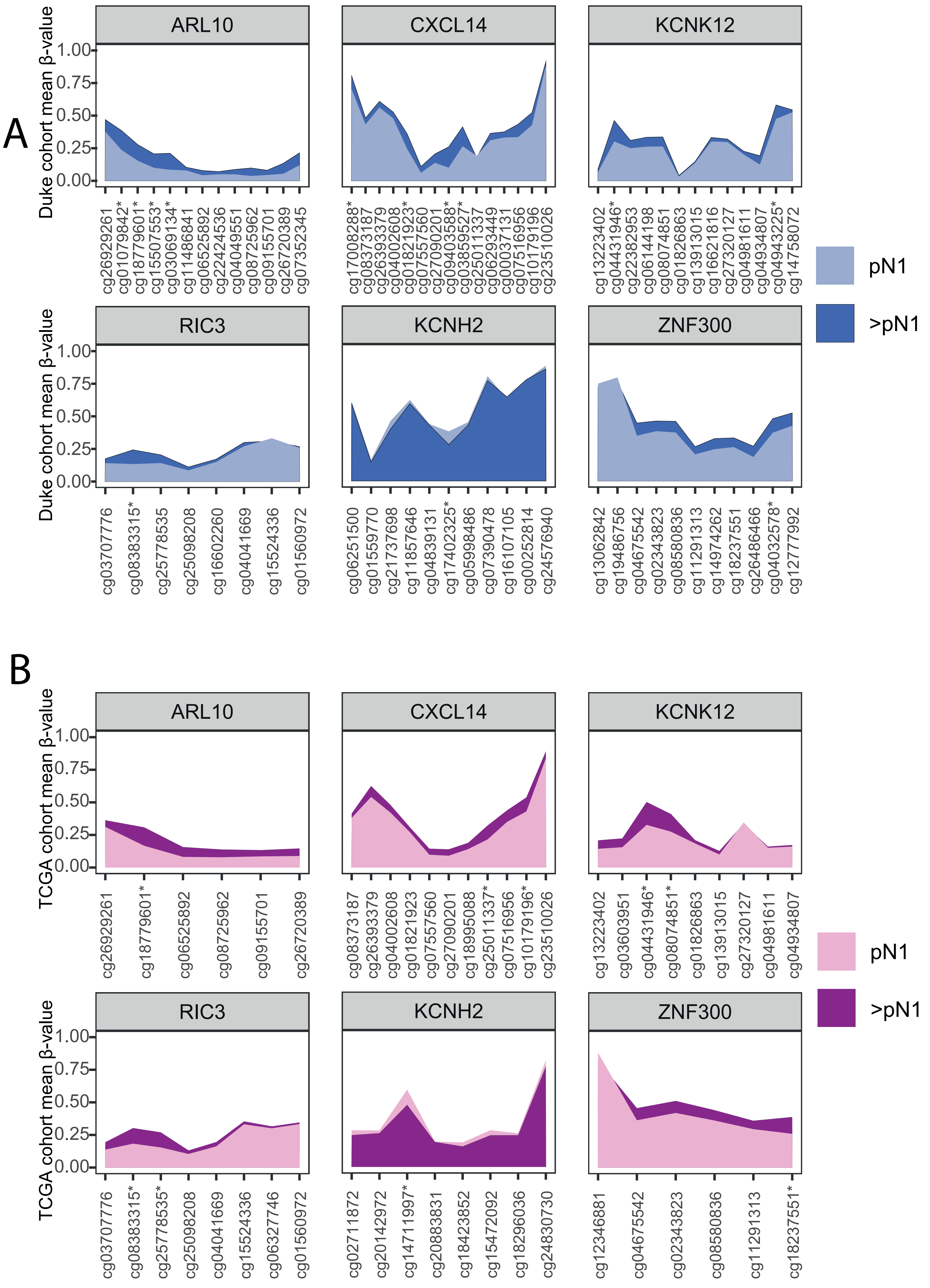
A
Duke cohort mean β-value
1.00
0.75
0.50
0.25
0.00
ARL10
cg26929261
cg01079842*
cg18779601*
cg15507553*
cg03069134*
cg11486841
cg06525892
cg22424536
cg04049551
cg08725962
cg09155701
cg26720389
cg07352345
CXCL14
cg17008288*
cg08373187
cg26393379
cg04002608
cg01821923*
cg07557560
cg27090201
cg09403588*
cg03859527*
cg25011337
cg06293449
cg00037131
cg07516956
cg10179196
cg23510026
KCNK12
cg13223402
cg04431946*
cg22382953
cg06144198
cg08074851
cg01826863
cg13913015
cg16621816
cg27320127
cg04981611
cg04934807
cg04943225*
cg14758072
pN1
>pN1
RIC3
cg03707776
cg08383315*
cg25778535
cg25098208
cg16602260
cg04041669
cg15524336
cg01560972
KCNH2
cg06251500
cg01559770
cg21737698
cg11857646
cg04839131
cg17402325*
cg05998486
cg07390478
cg16107105
cg00252814
cg24576940
ZNF300
cg13062842
cg19486756
cg04675542
cg02343823
cg08580836
cg11291313
cg14974262
cg18237551
cg26486466
cg04032578*
cg12777992
B
TCGA cohort mean β-value
ARL10
cg26929261
cg18779601*
cg06525892
cg08725962
cg09155701
cg26720389
CXCL14
cg08373187
cg26393379
cg04002608
cg01821923
cg07557560
cg27090201
cg18995088
cg25011337*
cg07516956
cg10179196*
cg23510026
KCNK12
cg13223402
cg03603951
cg04431946*
cg08074851*
cg01826863
cg13913015
cg27320127
cg04981611
cg04934807
pN1
>pN1
RIC3
cg03707776
cg08383315*
cg25778535*
cg25098208
cg04041669
cg15524336
cg06327746
cg01560972
KCNH2
cg02711872
cg20142972
cg14711997*
cg20883831
cg18423852
cg15472092
cg18296036
cg24830730
ZNF300
cg12346881
cg04675542
cg02343823
cg08580836
cg11291313
cg18237551*

Supplementary Figure S5. Promoter DNA methylation pattern of the six genes exhibiting concordant differences. (A) Duke cohort and **(B)** TCGA cohort**.** All DMS are indicated with an asterisk (*).

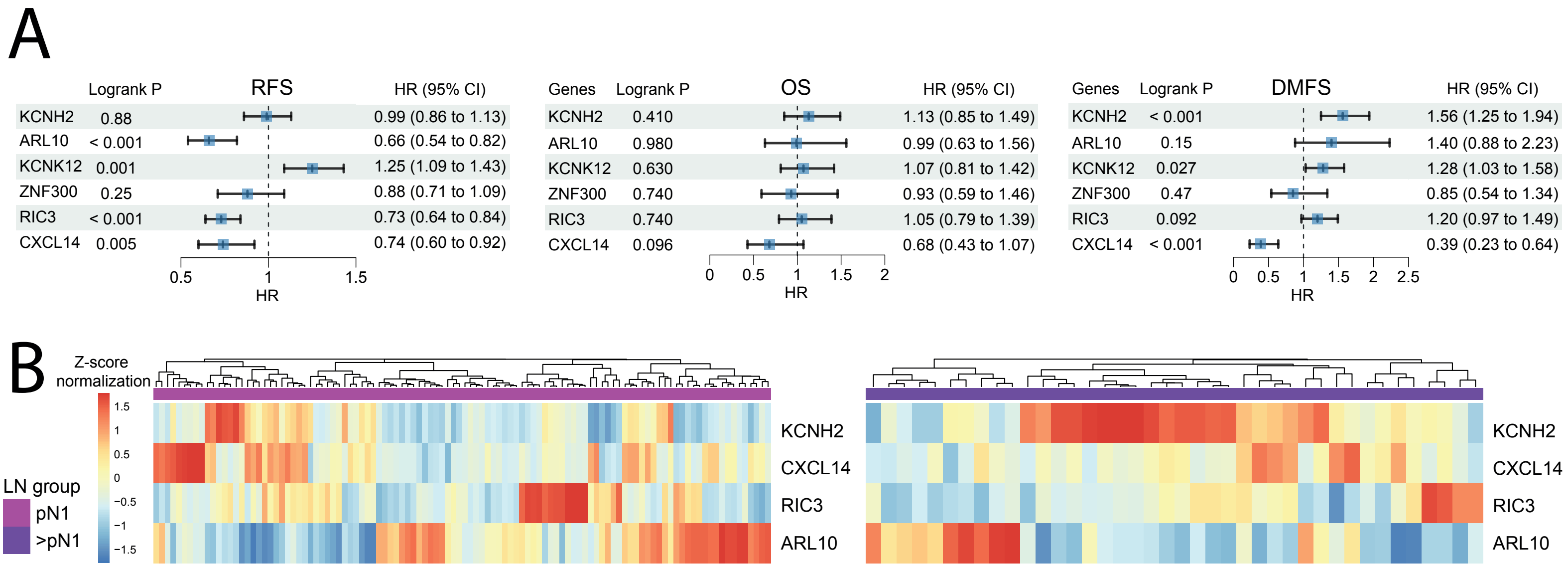
A
Logrank P
RFS
HR (95% CI)
KCNH2 0.88 0.99 (0.86 to 1.13)
ARL10 < 0.001 0.66 (0.54 to 0.82)
KCNK12 0.001 1.25 (1.09 to 1.43)
ZNF300 0.25 0.88 (0.71 to 1.09)
RIC3 < 0.001 0.73 (0.64 to 0.84)
CXCL14 0.005 0.74 (0.60 to 0.92)
0.5 1 1.5
HR
Genes
Logrank P
OS
HR (95% CI)
KCNH2 0.410 1.13 (0.85 to 1.49)
ARL10 0.980 0.99 (0.63 to 1.56)
KCNK12 0.630 1.07 (0.81 to 1.42)
ZNF300 0.740 0.93 (0.59 to 1.46)
RIC3 0.740 1.05 (0.79 to 1.39)
CXCL14 0.096 0.68 (0.43 to 1.07)
0 0.5 1 1.5 2
HR
Genes
Logrank P
DMFS
HR (95% CI)
KCNH2 < 0.001 1.56 (1.25 to 1.94)
ARL10 0.15 1.40 (0.88 to 2.23)
KCNK12 0.027 1.28 (1.03 to 1.58)
ZNF300 0.47 0.85 (0.54 to 1.34)
RIC3 0.092 1.20 (0.97 to 1.49)
CXCL14 < 0.001 0.39 (0.23 to 0.64)
0 0.5 1 1.5 2 2.5
HR
B
Z-score
normalization
1.5
1
0.5
0
−0.5
−1
−1.5
LN group
pN1
>pN1
KCNH2
CXCL14
RIC3
ARL10
KCNH2
CXCL14
RIC3
ARL10

Supplementary Figure S6. Survival associations and expression heterogeneity of the genes with concordant epigenetic-expression in ER+/HER2- BC (A) Forest plots depicting the association of six genes previously selected for concordant methylation and significant gene expression changes with relapse-free survival (RFS, left panel), overall survival (OS; middle panel), and distant metastasis-free survival (DMFS; right panel). **(B)** Heatmap of z-score–normalized expression of four genes with at least one significant clinical association in the TCGA dataset.

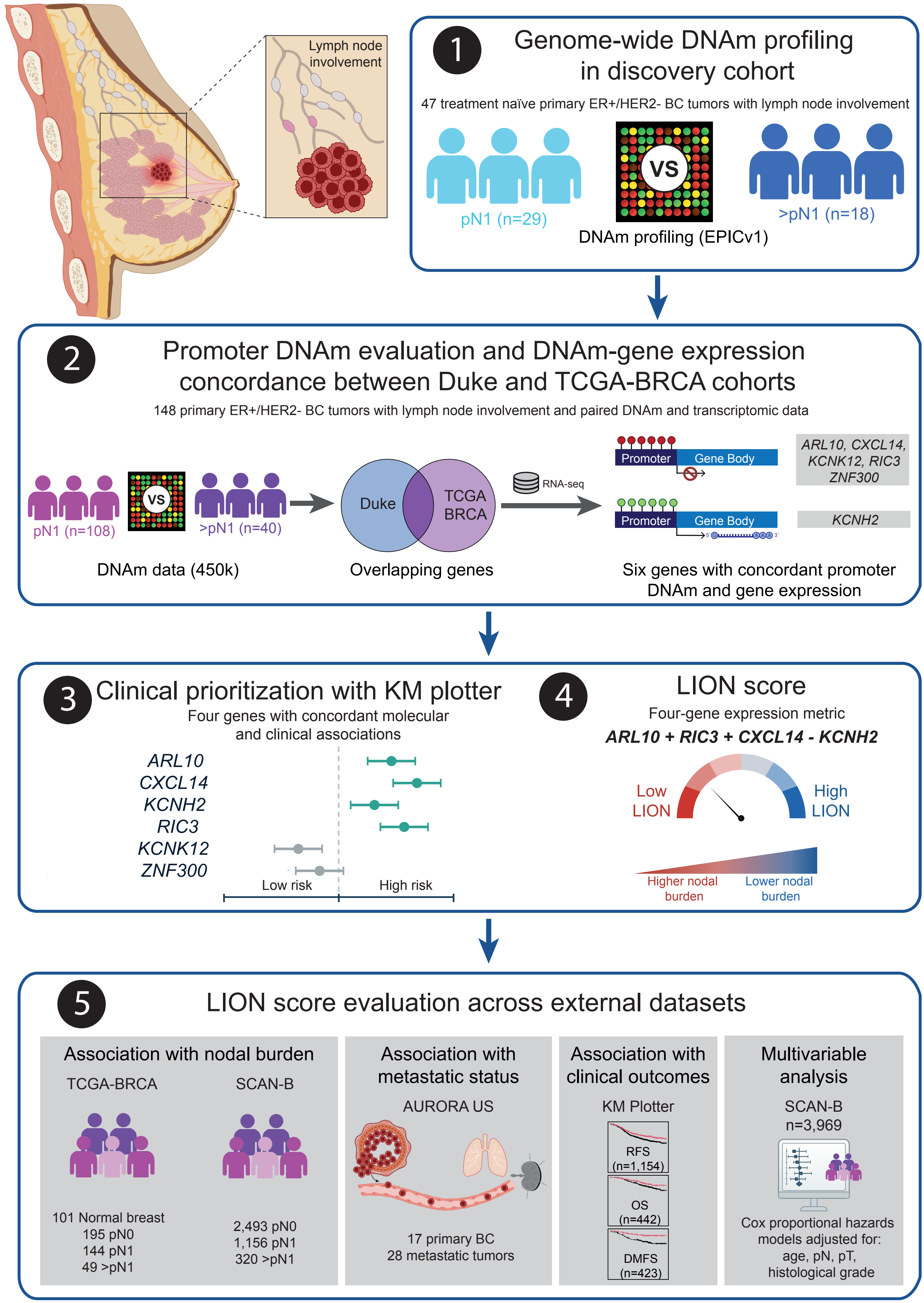
Lymph node involvement
1
Genome-wide DNAm profiling in discovery cohort
47 treatment naïve primary ER+/HER2- BC tumors with lymph node involvement
VS
pN1 (n=29)
>pN1 (n=18)
DNAm profiling (EPICv1)
2
Promoter DNAm evaluation and DNAm-gene expression concordance between Duke and TCGA-BRCA cohorts
148 primary ER+/HER2- BC tumors with lymph node involvement and paired DNAm and transcriptomic data
pN1 (n=108)
VS
>pN1 (n=40)
DNAm data (450k)
Duke
TCGA BRCA
Overlapping genes
RNA-seq
Promoter
Gene Body
ARL10, CXCL14, KCNK12, RIC3 ZNF300
KCNH2
Six genes with concordant promoter DNAm and gene expression
3
Clinical prioritization with KM plotter
Four genes with concordant molecular and clinical associations
ARL10
CXCL14
KCNH2
RIC3
KCNK12
ZNF300
Low risk
High risk
4
LION score
Four-gene expression metric
ARL10 + RIC3 + CXCL14 - KCNH2
Low LION
High LION
Higher nodal burden
Lower nodal burden
5
LION score evaluation across external datasets
Association with nodal burden
TCGA-BRCA
SCAN-B
101 Normal breast
195 pN0
144 pN1
49 >pN1
2,493 pN0
1,156 pN1
320 >pN1
Association with metastatic status
AURORA US
17 primary BC
28 metastatic tumors
Association with clinical outcomes
KM Plotter
RFS (n=1,154)
OS (n=442)
DMFS (n=423)
Multivariable analysis
SCAN-B
n=3,969
Cox proportional hazards models adjusted for: age, pN, pT, histological grade

Supplementary Figure S7. Study design and external evaluation strategy. Schematic overview of the discovery, molecular integration, LION score derivation, and external evaluation workflow across the Duke, TCGA-BRCA, KM Plotter, SCAN-B, and AURORA US datasets.
